# Using bicistronic constructs to evaluate the chaperone activities of heat shock proteins in cells

**DOI:** 10.1038/s41598-017-02459-9

**Published:** 2017-05-24

**Authors:** Rebecca San Gil, Tracey Berg, Heath Ecroyd

**Affiliations:** 1 Illawarra Health and Medical Research Institute, University of Wollongong, Northfields Ave, Wollongong, 2522 Australia; 2 School of Biological Sciences, University of Wollongong, Northfields Ave, Wollongong, 2522 Australia

## Abstract

Heat shock proteins (Hsps) are molecular chaperones that prevent the aggregation of client proteins by facilitating their refolding, or trafficking them for degradation. The chaperone activities of Hsps are dependent on dynamic protein-protein interactions, including their oligomerisation into large multi-subunit complexes. Thus, tagging Hsps with fluorescent proteins can interfere with their chaperone activity. To overcome this limitation, we have exploited bicistronic constructs for the concurrent expression of a non-tagged Hsp and fluorescent reporter from a single mRNA in cells. We used the Hsp-encoding bicistronic constructs in a cell-based model of protein aggregation, using a destabilised (mutant) form of firefly luciferase (mFluc) that forms inclusion bodies in cells. Expression of Hsp40, Hsp70, or Hsp40 and Hsp70 in cells expressing mFluc decreased the formation of inclusion bodies by 25–46% compared to controls. Moreover, there was a concentration-dependent decrease in the proportion of cells with inclusions when Hsp70, or Hsp40 and Hsp70 were co-expressed with mFluc in cells. The Hsp-encoding bicistronic constructs enable transfection efficiencies and concentration-dependent effects of Hsp expression to be determined using fluorescence based techniques, without the need to tag the Hsp with a fluorescent protein.

## Introduction

Neurodegenerative diseases, such as Alzheimer’s disease, Parkinson’s disease and amyotrophic lateral sclerosis, are characterised by the deposition of misfolded and aggregated proteins in specific regions of the brain and spinal cord^1–3^. The initiation of protein aggregation in these diseases is due to, at least in part, the dysfunction in the proteostasis network^4^. The cytotoxic mechanism(s) associated with pathogenic protein aggregation is largely unknown, however, it can be partially explained by aberrant interactions between aggregates and other proteins involved in key cellular pathways^5^.

Molecular chaperones are a central component of the proteostasis network as they facilitate the correct folding of nascent polypeptides, maintain misfolded proteins in folding-competent states, re-fold damaged proteins, and shuttle destabilised proteins for degradation by the proteasome or autophagy^6^. A recent and comprehensive analysis of the human “chaperome” identified 332 chaperone genes, 147 of which correspond to the heat shock protein subfamilies Hsp90, Hsp70, Hsp60, Hsp40 and small Hsps (sHsps)^7^. The Hsps are a family of evolutionarily conserved chaperones with diverse functions and molecular mechanisms of action. For example, members of the Hsp90, Hsp70 (in conjunction with its co-chaperone Hsp40) and Hsp60 families are ATP-dependent “foldases” that prevent protein aggregation by re-folding damaged or misfolded proteins back to their native state^8–11^. The sHsps are ATP-independent and often referred to as “holdases” or “stabilisers” as a result of their ability to maintain misfolded proteins in folding-competent states, which facilitates refolding by foldases^12^. Heat shock proteins are endogenously expressed in some cells for ‘house-keeping’ roles (e.g. Hsp27 is important in cytoskeletal actin regulation)^13^, however, under conditions of cellular stress, their levels can be dramatically up-regulated to further stabilise the cytoskeleton, regulate stress responses, and mitigate apoptotic signalling^12, 14^. Collectively, these functions make Hsps attractive targets for the development of therapeutics that can modulate the underlying molecular mechanisms that cause neurodegeneration.

Previous work has demonstrated that Hsps can prevent the disease-associated aggregation of proteins and the toxicity associated with this process in cells. For example, Ormsby *et al*.^15^ showed, by flow cytometric pulse shape analysis, that Hsp40 inhibited the aggregation of pathogenic polyglutamine-expanded huntingtin^15^. In contrast, whilst Hsp70 reduced cell death in this model, it had no effect on inclusion body formation^15^. However, the relative co-transfection efficiencies and levels of the Hsp in cells cannot be easily determined when the expressed Hsps are not fluorescently tagged. In particular, this confounds efforts to compare the effect of different Hsps on cellular functions. Vos *et al*.^16^ performed a systematic comparison of the chaperone efficacy of human sHsp family members in inhibiting polyglutamine-expanded huntingtin in cells, in which immunoblotting was performed to determine the relative expression levels of each sHsp in HEK293 cells (each sHsp had a C-terminal V5-tag to enable detection with the same anti-V5-antibody)^16^. In this work, the expression levels of HspB7 and HspB9 were significantly lower than the expression levels of the other sHsps investigated. However, immunoblotting did not reveal whether the differences in expression levels were attributable to a lower rate of expression, a lower transfection efficiency of the HspB7 and HspB9-encoding constructs compared to the other constructs used, or higher turn-over rate of HspB7 and HspB9 in these cells^16^. Moreover, such techniques do not provide any information regarding the levels of the expressed protein in individual cells. Thus, it is advantageous to be able to account for transfection efficiencies and the levels of Hsps in cell-based assays, particularly when the aim is to compare the activities of different Hsps.

The functions of many proteins have been studied by tagging them to a fluorescent protein. However, Hsps are dynamic (and often oligomeric) proteins that interact with various co-factors and client proteins. For example, some sHsps, such as αB-crystallin (αB-c) and Hsp27, form large and polydisperse homo- and hetero-oligomers with other sHsps, and undergo dynamic subunit exchange, features that are thought to be fundamental to their chaperone activity^17, 18^. Therefore, the addition of a fluorescent tag can compromise their activity. This has been demonstrated by a recent study that investigated the function of recombinant forms of Hsp27 and αB-c and showed that labelling their N- or C-termini with fluorescent proteins (i.e. green fluorescent protein derivatives) severely affected their oligomeric assembly and subsequent chaperone activity compared to non-tagged proteins^19, 20^. Labelling the N-terminus of Hsp27 with enhanced yellow fluorescent protein resulted in the formation of small, unstable oligomers (5–9 subunits) compared to wild-type Hsp27, which forms large, stable oligomers consisting of more than 20 subunits^21^. Since the physiochemical properties of fluorescently labelled Hsps have not been fully elucidated in cells, and fluorescently labelled Hsps can show aberrant structure and function compared to the non-tagged protein, an alternative technique is needed for studying Hsp function in cells.

Given these limitations, there is a clear need to develop strategies to evaluate and compare Hsp functions in cells that take into account differences in transfection efficiencies, and avoid the use of bulky fluorescent proteins to label them. With this in mind, we have exploited bicistronic vectors to develop a suite of mammalian expression constructs for the correlated expression of non-labelled Hsps and a fluorescent reporter protein (e.g. enhanced green fluorescent protein, EGFP, or mCherry). The Hsp-encoding constructs generate bicistronic mRNA with an internal ribosomal entry site (IRES) between the multiple cloning site and the fluorescent reporter gene^22^. Translation of this mRNA results in the expression of two separate proteins, the Hsp of interest and the fluorescent reporter.

Using these Hsp-encoding bicistronic constructs, the capacity of a range of Hsps (Hsp90, Hsp70, Hsp40, Hsp27, and αB-c) to inhibit protein aggregation in a cell-based model were evaluated. The development of these bicistronic constructs provides a useful new tool to evaluate the role of Hsps in the proteostasis network and their capacity to modulate a range of key cellular processes. This strategy also has applications beyond the field of proteostasis, for example, the study of proteins in cells in which labeling with a fluorescent protein is not a viable option.

## Results

### Validation of correlated Hsp and fluorescent reporter expression from bicistronic constructs

We sought to develop a technique to study the ability of Hsps to prevent protein aggregation in live cells that avoided some of the limitations of previous work (i.e. tagging Hsps with fluorescent proteins which can affect structure, dynamics and function; inability to take into account differences in co-transfection efficiencies between plasmids; lack of information regarding the expression levels of the Hsps in individual cells). An experimental set-up where the Hsp and a fluorescent reporter protein are expressed from two separate plasmids is one possible approach. Figure S1 shows that cells co-transfected to express mCherry and EGFP do so in a correlated manner. However, in order to simplify the experimental design, we considered the use of bicistronic vectors for the simultaneous expression of an Hsp and fluorescent reporter from a single construct. Since both proteins are translated from the same mRNA transcript, the transcription of which is driven by a single promoter, this approach has the potential to overcome the need to transfect with an additional plasmid.

**Figure 1:**
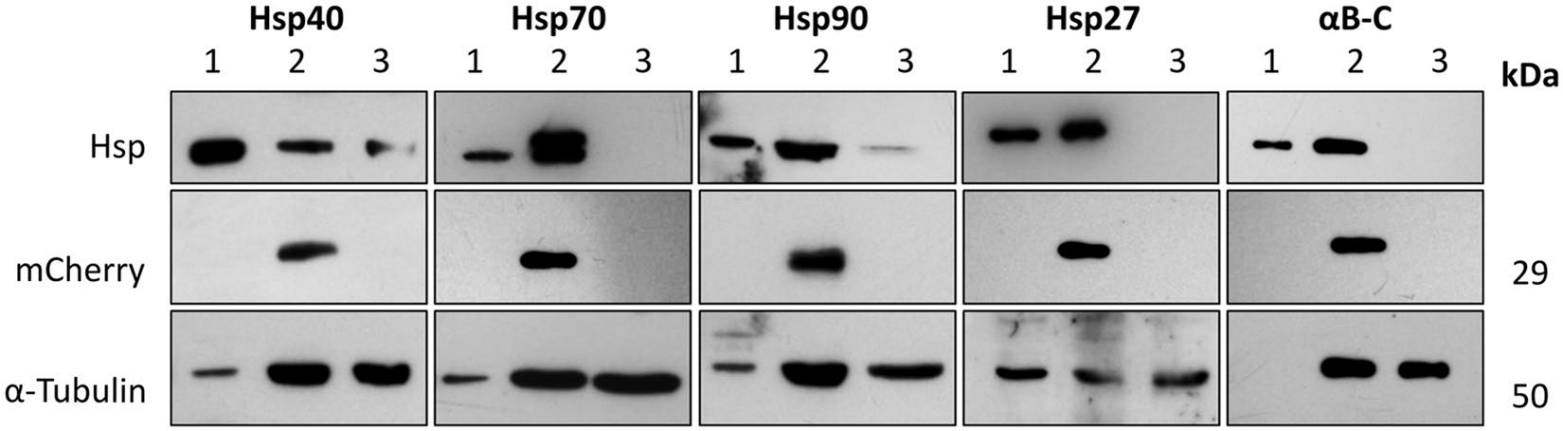
Immunoblot analysis of Hsp and mCherry expression in Neuro-2a cells transfected with one of the Hsp-encoding pIRES2 bicistronic constructs. Neuro-2a cells transfected with one of the Hsp-encoding bicistronic constructs were sorted by FACS such that a population of mCherry^+ve^ cells were purified and whole cell lysates equivalent to 100,000 cells were loaded into each well. The membranes were probed for α-tubulin (50 kDa), mCherry (29 kDa) and Hsp40, Hsp70, Hsp90, Hsp27, and αB-c. Protein samples analysed were (1) positive control sample consisting of either 10 µg of heat-shocked HeLa cell lysate (42 °C, 2 h with a 37 °C, 3 h recovery period) or 5 ng purified recombinant αB-c for blots probing for αB-c, (2) whole cell lysates from cells transfected with the corresponding Hsp-encoding bicistronic construct, and (3) untransfected cells.

Immunoblot analysis was performed to confirm the over-expression of the Hsp of interest and fluorescent reporter (in this case mCherry) in Neuro-2a cells transfected with one of the Hsp-encoding pIRES2-mCherry constructs. Immunoblot analysis of cells transfected with each of the pIRES2-mCherry constructs encoding for Hsp40, Hsp70, Hsp90, Hsp27 and αB-c showed that the Hsp and mCherry were expressed in these cells (Fig. 1). With the exception of Hsp40 and Hsp90, no endogenous Hsp expression was detected in untransfected Neuro-2a lysates (Figs 1 and S2).

**Figure 2:**
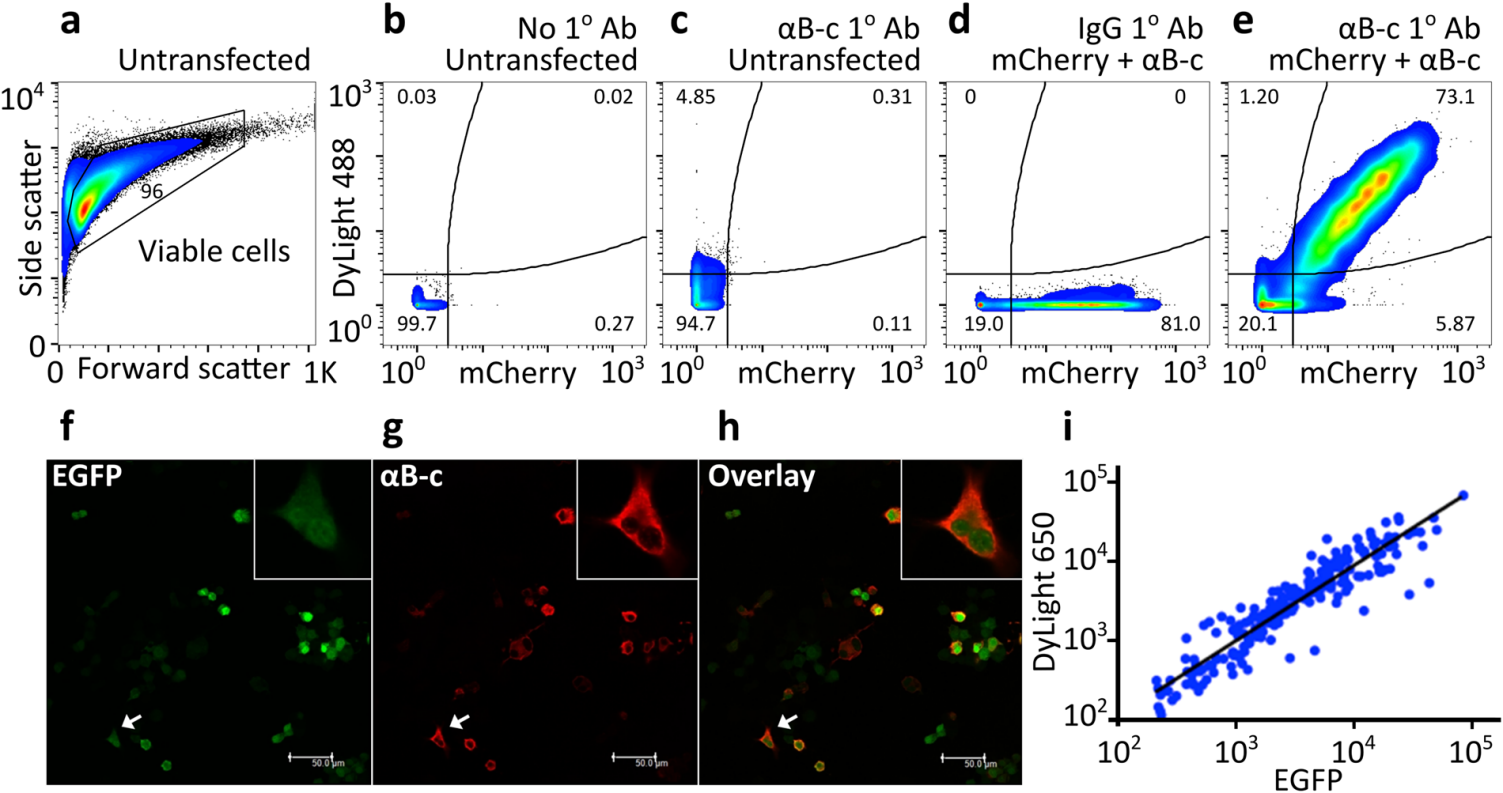
Validating the correlated expression of an Hsp and fluorescent reporter protein in transfected Neuro-2a cells. (**a–e**) Flow cytometric analysis of αB-c and mCherry protein expression in pIRES2-mCherry- αB-c transfected Neuro-2a cells. Data are presented as pseudo-colour plots where blue depicts – low, green – medium and red – high frequency of cells. Outliers are shown as black dots. (**a**) The untransfected sample was used to set gates for the viable cell population to exclude cellular debris and cell clumps. (**b**) Quadrant gating of DyLight 488 and mCherry fluorescence was based on untransfected and unlabeled cells. (**c**) Untransfected cells were immunolabelled with anti-αB-c and DyLight 488-conjugated secondary antibodies. Cells transfected with pIRES2-mCherry-αB-c were immunolabelled with (**d**) species-matched IgG isotype control antibody to determine background staining and (**e**) anti-αB-c and DyLight 488 conjugated secondary antibodies. (**f**–**i**) Immunofluorescence microscopy to analyse the expression of αB-c and EGFP following transfection of Neuro-2a cells with pIRES2-EGFP-αB-c. Intracellular αB-c was immunolabelled using anti-αB-c primary and anti-mouse IgG DyLight 650 conjugates secondary antibodies. Coverslips were mounted onto glass slides and emissions from (**f**) EGFP and (**g**) DyLight 650 were acquired. (**h**) The overlay of EGFP and DyLight 650 emissions is also presented. Insets show magnification of the cell identified by the arrow. Scale bar = 50 µm. (**i**) EGFP and DyLight 650 fluorescence levels of individual cells.

We next tested for the correlated expression of the non-labeled Hsp and fluorescent reporter from each of the bicistronic constructs in cells, since this would enable the fluorescence intensity of the fluorescent protein to be used as a reporter of intracellular Hsp levels. Neuro-2a cells were transfected with an Hsp-encoding bicistronic construct and intracellular Hsps were immunolabelled with specific primary and DyLight 488 (or in the case of the pIRES2-EGFP-Hsp constructs DyLight 650)-conjugated secondary antibodies and subsequently analysed by flow cytometry and confocal microscopy (Fig. 2).

To exclude cellular debris and cell clumps from the flow cytometric analysis, a polygonal gate was used to identify viable cells based on a plot of forward and side scatter (Fig. 2a). Quadrant gating based on the untransfected and unlabeled sample (i.e. DyLight 488^−ve^: mCherry^−ve^) was used to establish background fluorescence (Fig. 2b). Low levels of DyLight 488 fluorescence were observed in untransfected cells immunolabelled for αB-c, indicating that these cells express low levels of endogenous αB-c (Fig. 2c). Samples incubated with an isotype (IgG) species-matched control primary antibody exhibited no DyLight 488 fluorescence, confirming no non-specific binding had occurred in the labeling process (Fig. 2d). Cells transfected with the pIRES2-mCherry-αB-c construct were positive for both DyLight 488 and mCherry fluorescence (Fig. 2e). Likewise, levels of Hsp27 and Hsp70 correlated well with levels of mCherry fluorescence in transfected cells (Fig. S3). Whilst the levels of Hsp40 and Hsp90 and fluorescent reporter were correlated in cells, this correlation was weaker than for the other Hsps tested (Fig. S3). This may be due to Hsp40 and Hsp90 both being endogenously expressed in Neuro-2a cells, whereas Hsp27, αB-c and Hsp70 are not expressed at levels detectable by immunoblotting in untransfected Neuro-2a cells (Fig. 1). Similarly, confocal microscopy of Neuro-2a transfected with pIRES2-EGFP-αB-c also demonstrated a strong correlation between the expression of αB-c and EGFP reporter protein (Fig. 2f–i). Confocal microscopy also showed that αB-c and EGFP were not fused because EGFP was localized in the nucleus and cytoplasm, whereas αB-c was only observed in the cytoplasm.

### Cell-based mFLuc-EGFP aggregation assay

To assess the relative ability of each Hsp to prevent the aggregation of proteins into inclusions in cells, a conformationally destabilised form of firefly luciferase, C-terminally tagged with EGFP (R188Q/R261Q; mFLuc-EGFP), was used as an aggregation-prone protein^23^. This isoform of firefly luciferase has previously been shown to form cytosolic inclusion bodies in HeLa cells when cultured at 37 °C^23^. Importantly, in these co-transfection experiments, the mFluc-EGFP-encoding plasmid and the Hsp-encoding IRES plasmid were added separately to cells (i.e. each DNA:lipid complex was made up separately and then added to the cells, instead of mixing the two plasmids together prior to making the DNA:lipid complexes). We transfected the cells in this way because when the plasmids are first mixed and then DNA:lipid complexes are made and applied to cells there is a very strong correlation in the expression of proteins from both plasmids (Fig. S1) such that the majority of cells that express high levels of mFluc-EGFP also express high levels of the Hsp. Making separate DNA:lipid complexes for both plasmids and then adding these to cells resulted in a greater range in the relative expression of proteins from both plasmids in the population (i.e. a range of levels of Hsp expression at a given level of mFluc-EGFP expression; Fig. S6).

**Figure 3:**
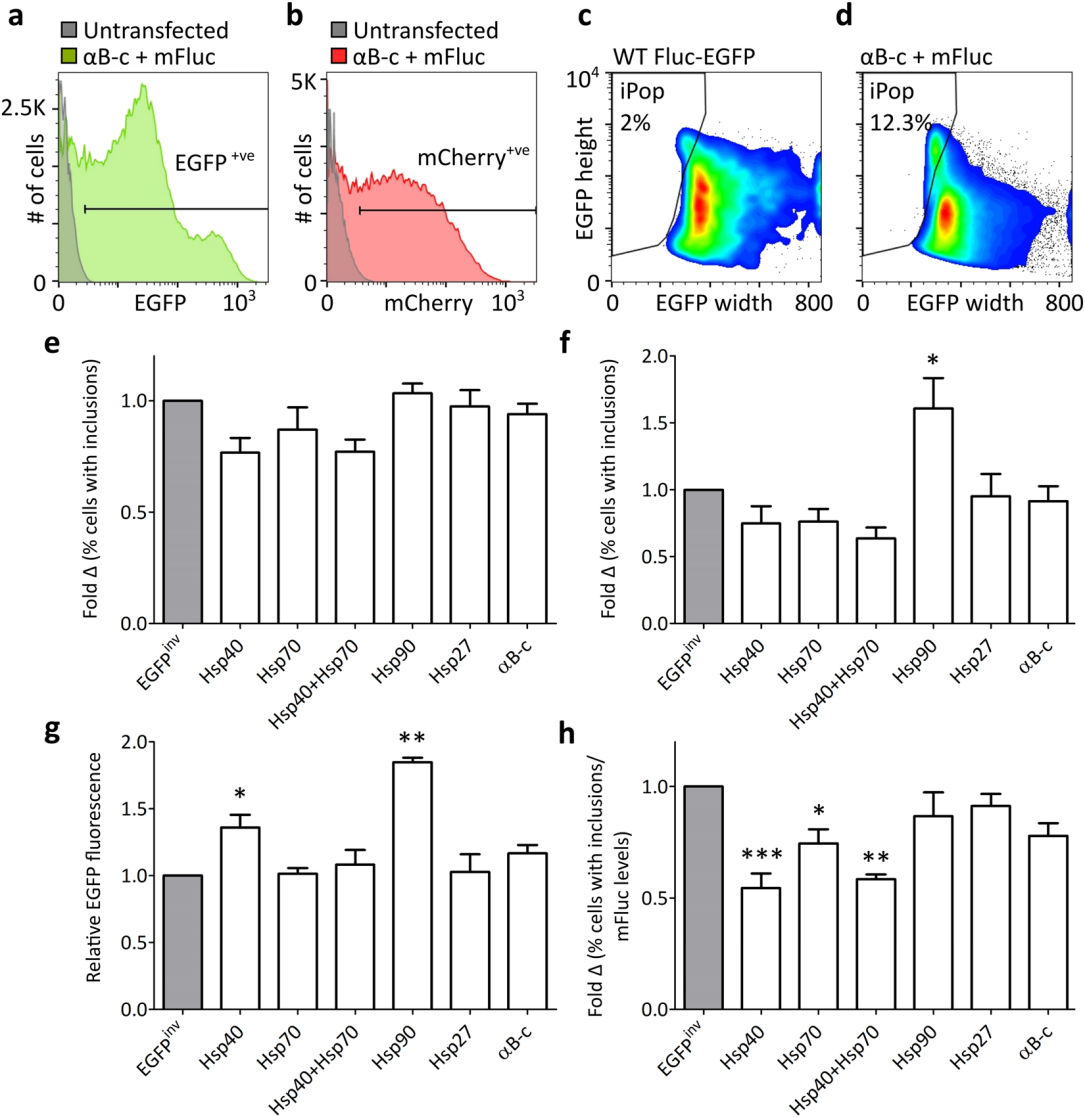
Using Hsp-encoding bicistronic constructs to examine the effect of Hsp over-expression on mFluc-EGFP inclusion body formation in cells. (**a**–**d**) Gating strategy employed to analyse flow cytometric data from Neuro-2a cells co-transfected to express mFluc-EGFP and one of the Hsps (or a control protein, EGFP^inv^) with a mCherry reporter. Untransfected cells were used to set gates for (**a**) EGFP^+ve^ and (**b**) mCherry^+ve^ cells. Representative samples co-transfected with the pIRES2-mCherry-αB-c and mFluc-EGFP constructs are shown in the histogram overlays with the untransfected sample in (**a**) and (**b**). Cells expressing WT Fluc-EGFP (**c**) were used to set the iPop gate and (**d**) the population of viable, EGFP^+ve^ and mCherry^+ve^ cells with mFluc-EGFP inclusions (iPop) was resolved by pulse shape analysis using plots of EGFP fluorescence height versus width. (**e**–**h**) Show a comparison of three strategies used to analyse the proportion of cells containing mFluc-EGFP inclusion bodies in the iPop. (**e**) The proportion of cells with mFluc-EGFP inclusions when only viable and EGFP^+ve^ cells were taken into account. (**f**) The proportion of cells with mFluc-EGFP inclusions when viable and co-transfected cells (i.e. EGFP^+ve^ and mCherry^+ve^) were analysed. (**g**) The average relative EGFP fluorescence in cells as a measure of mFluc-EGFP expression levels. (**h**) The proportion of cells with mFluc-EGFP inclusions normalised to the levels of mFluc-EGFP in cells (based on relative EGFP fluorescence levels). In all graphs, data are reported as the fold difference relative to the chaperone-negative control, EGFP^inv^. Data presented are the means + SEM of three biological replicates. The raw percent of cells in the iPop in each biological replicate is shown in Fig. S7. Statistically significant differences between the means were assessed using a one-way ANOVA followed by a Dunnett’s post-hoc test (*p < 0.05, **p < 0.01, ***p < 0.001).

Neuro-2a cells were co-transfected with one of the Hsp-encoding bicistronic constructs and the mFLuc-EGFP-encoding construct, and the cells incubated for 48 h prior to analysis by flow cytometry. Cellular debris and cell clumps were excluded from subsequent analyses using forward and side scatter signals (as in Fig. 2a). The untransfected sample was used as an EGFP^−ve^ and mCherry^−ve^ population to identify EGFP^+ve^ and mCherry^+ve^ cells (Fig. 3a and b). Sub-populations of cells with mFluc-EGFP inclusion bodies were detected using flow cytometry-based pulse shape analysis (inclusion population - iPop; non-inclusion population - niPop)^20^.PulSA can resolve populations of cells with fluorescent inclusions when this leads to a change in the fluorescent pulse-shape of the cell (reduced fluorescent pulse width and increased fluorescent pulse height) compared to cells lacking inclusions^21^. We confirmed, by cell sorting and imaging, that cells in the iPop contained mFluc-EGFP inclusions whereas the vast majority of those in the niPop did not contain inclusions (Fig. S4). Analysing cells via PulSA demonstrated that only a minor (2%) proportion of cells expressing the stable wild-type Fluc-EGFP isoform contained inclusions (Fig. 3c), whereas the proportion of cells containing inclusions increased (to >10%) when they expressed mFluc-EGFP (Fig. 3d).

In order to assess the impact Hsps had on the proportion of cells with inclusions, three methods were employed to analyse the data (Fig. 3e,f and h). First, only cells expressing mFluc-EGFP were taken into account (Fig. 3e), a strategy that is indicative of the type of data analysis that is performed in assays where the Hsp is not fluorescently labeled. This approach demonstrated that there was a significant effect of over-expressing different Hsps on the proportion of cells with mFluc-EGFP inclusions [F(6, 14) = 3.011, *P* = 0.0418]. There was a small reduction in the proportion of cells with mFluc-EGFP inclusions that were co-transfected to express Hsp40, Hsp70 or Hsp40 + Hsp70, compared to those cells co-transfected to express EGFP^inv^, however, post hoc comparisons using Dunnett’s test showed that these differences were not statistically significant. In contrast, expression of Hsp90, Hsp27 or αB-c had a negligible effect on the proportion of cells with mFluc-EGFP inclusions (Fig. 3e). When an alternative analysis strategy that takes into account differences in co-transfection efficiencies between the Hsp-encoding constructs was applied (i.e. analysis of EGFP^+ve^ and mCherry^+ve^ cells; Figs 3f and Fig. S5), one-way ANOVA indicated there is a significant effect of Hsp expression on mFluc-EGFP inclusion formation [F(6, 14) = 5.739, *P* = 0.0034]. There was a significant (161 ± 23%) increase in the proportion of cells with mFluc-EGFP inclusions in cells co-expressing Hsp90, compared to those co-expressing EGFP^inv^. The expression of Hsp40, Hsp70, or Hsp40 and Hsp70 resulted in a small (but not statistically significant) decrease in the proportion of cells with mFluc-EGFP inclusions, compared to those expressing EGFP^inv^. Expression of Hsp27 or αB-c had a negligible effect on the proportion of cells with inclusions.

Previous studies have demonstrated that high levels of expression of aggregation-prone proteins are strongly correlated with an increased propensity for inclusion body formation^15, 24, 25^. We therefore examined whether there were differences in the levels of mFLuc-EGFP expressed in cells upon co-transfection with the Hsp-encoding constructs (Fig. 3g). One-way ANOVA analysis demonstrated that the median EGFP fluorescence intensity varied significantly between samples co-expressing different Hsps [F(6,14) = 14.46, *P < *0.0001]. Co-transfection with the Hsp40 (135 ± 9%) or Hsp90 (185 ± 3%) bicistronic constructs resulted in a significant increase in the levels of mFLuc-EGFP in co-transfected cells compared to those co-transfected to express EGFP^inv^. When these differences in the relative levels of mFluc-EGFP expression were taken into account in analyzing the aggregation-propensity of mFluc in cells (Fig. 3h) it was found that there was a significant effect of Hsp expression on the proportion of cells with mFLuc-EGFP inclusions [F(6,14) = 7.475, *P* = 0.0010]. The expression of Hsp40, Hsp70 or Hsp40 + Hsp70 significantly reduced the proportion of cells with mFLuc-EGFP inclusions (46 ± 7%, 26 ± 6%, and 42 ± 2% reduction, respectively) compared to the cells expressing EGFP^inv^. Whilst the expression of Hsp90, Hsp27 or αB-c reduced the proportion of cells with mFLuc-EGFP inclusion bodies, this was not statistically significant.

**Figure 4:**
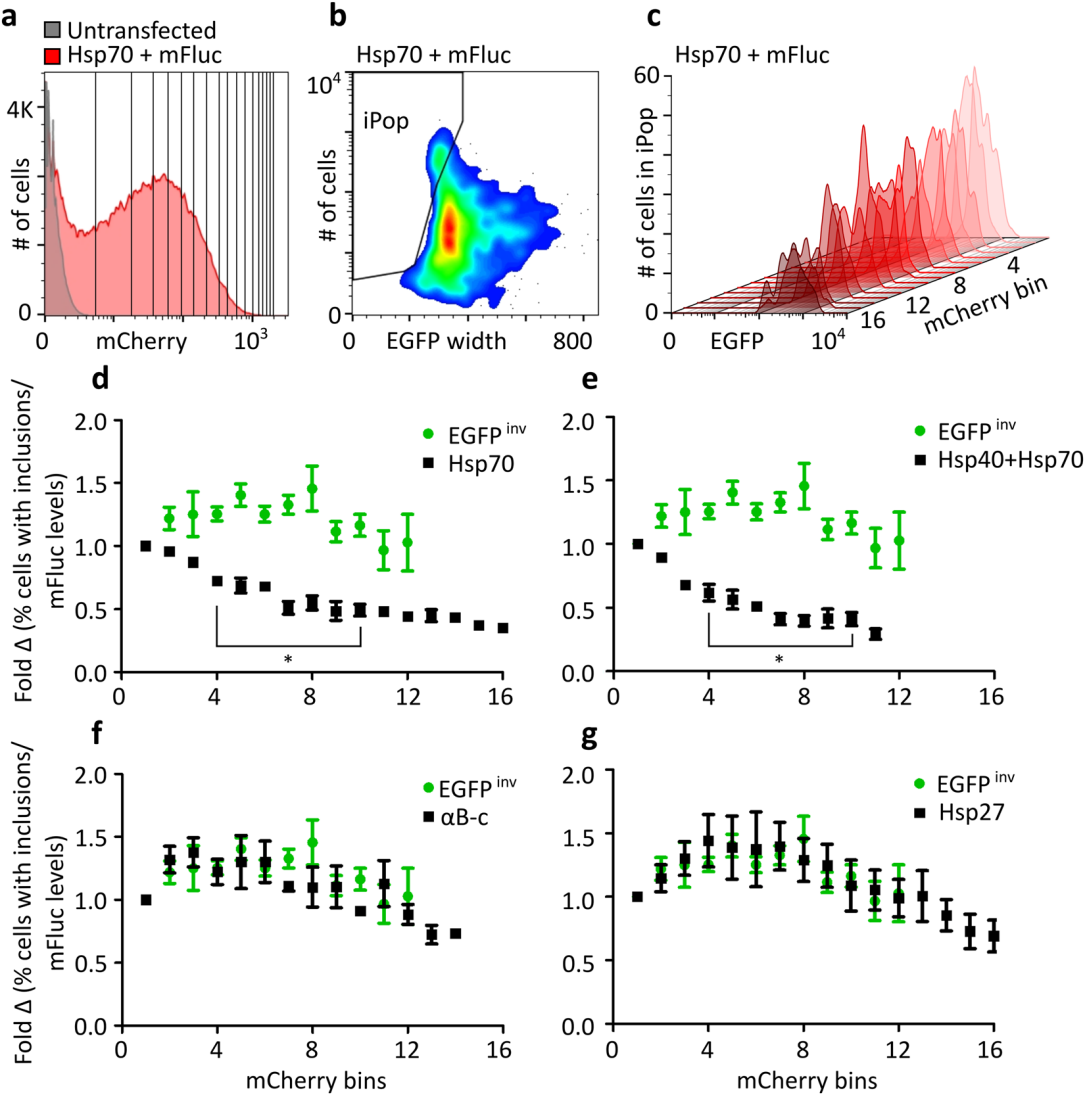
Use of bicistronic constructs to determine the effect of increasing Hsp levels on the formation of mFluc-EGFP inclusion bodies in cells. (**a**) Gating strategy used to determine the fraction of cells with inclusion bodies as a function of mCherry (Hsp) expression. The frequency histogram of mCherry fluorescence was subdivided into 16 bins of equal mCherry RFU (40 RFU), where bin 1 represents the lowest, and bin 16 the highest level of mCherry expression. Pulse shape analysis was used to obtain (**b**) the proportion of cells with inclusions in each mCherry bin. (**c**) The frequency histogram overlay shows the relative reduction in the number of cells with inclusions with increasing mCherry reporter protein, using the mCherry binning strategy. Neuro-2a co-transfected to express Hsp70 with mCherry and mFluc-EGFP are shown in these representative plots. (**d,g**) Fold change in cells with inclusions as a function of mCherry fluorescence (normalised to mFluc-EGFP levels) when transfected with EGFP^inv^ and (**d**) Hsp70, (**e**) Hsp40 and Hsp70, (**f**) αB-c, and (**g**) Hsp27-encoding bicistronic constructs. Mean ± SEM of three biological replicates. Statistically significant differences between the means of cells expressing EGFP^inv^ and Hsp were assessed at each mCherry bin using a student’s t-test where *p < 0.05 was considered significant. mCherry bins with less than 100 events were excluded from subsequent analysis.

We next exploited the correlation between the levels of Hsps and the fluorescent reporter in cells in order to investigate the effect of increasing Hsp levels on the proportion of cells with mFluc-EGFP inclusions. To do so, cells co-expressing an Hsp (or EGFP^inv^ control) and mFluc-EGFP, were sub-divided into “bins” of equal width (40 RFU) based on the level of mCherry fluorescence (the Hsp reporter; Figs 4a and S5). Pulse shape analysis was then used to determine the proportion of cells with inclusions in each of these bins. The relative mFluc-EGFP expression levels (i.e. the EGFP fluorescent median) in each bin were used to normalize the proportion of cells in the iPop of each respective mCherry bin (Fig. 4b). For cells co-transfected to express Hsp70 and mFluc, increasing levels of Hsp70 resulted in a significant decrease in the proportion of cells with mFluc-EGFP inclusions compared to cells expressing EGFP^inv^ (Fig. 4c,d). Similarly, increasing levels of Hsp40 + Hsp70 resulted in a significant reduction in the proportion of cells with mFluc-EGFP inclusions, relative to cells expressing EGFP^inv^ (Fig. 4e). This indicates that these Hsps work in a concentration-dependent manner. Conversely, increasing concentrations of αB-c and Hsp27 had no effect on the proportion of cells with mFluc-EGFP inclusions (Fig. 4f,g). Cells expressing Hsp40 and Hsp90 were excluded from this type of analysis due to the weaker correlation observed between levels of the Hsp and the reporter protein (Fig. S3).

## Discussion

The dynamic and complex nature of the interactions Hsps have with themselves and their client proteins presents a barrier to studying them in cells using fluorescent tags. To overcome this, we have developed and validated a suite of Hsp-encoding bicistronic constructs for the correlated expression of a non-tagged Hsp and a fluorescent reporter protein. These bicistronic constructs can be used to study and compare the cytoprotective functions and chaperone abilities of Hsps in the physiologically relevant context of the cell.

Before using these bicistronic constructs to evaluate the function of Hsps in cell-based assays, we validated the correlated expression of the Hsp and fluorescent reporter protein. A strong correlation between the Hsps and fluorescent reporter protein was observed in cells transfected with αB-c, Hsp27 or Hsp70-encoding bicistronic constructs, which corresponded to Hsps that were found not to be endogenously expressed in Neuro-2a cells (as determined by immunoblotting of cell lysates). The correlation between the levels of Hsp40 or Hsp90 and the fluorescent reporter protein were not as strong. It is not currently clear why this is the case. It is possible that the endogenous expression of Hsp90 and Hsp40 observed in Neuro-2a, combined with over-expression from the bicistronic construct, may promote a higher turn-over of these Hsps in these cells. Interestingly, the level of the mCherry reporter from the Hsp40-encoding IRES vector was also significantly lower than observed from the other Hsp-encoding constructs (see Fig. S2) suggesting that there are differences in the levels of transcription and translation from these constructs, which is dependent on the genes being expressed. Whatever the reason, through using the bicistronic constructs we were able to take into consideration differences in transfection efficiency and protein expression levels in downstream analyses. Together, these data emphasise the importance of validating the correlated expression of proteins expressed from bicistronic constructs, since relative levels may vary significantly between constructs.

In previous studies that have expressed Hsps without a fluorescent tag in cells, differences in transfection and co-transfection efficiencies between samples could not easily be taken into account in downstream analyses. In contrast, the use of Hsp-encoding bicistronic constructs enables transfection efficiencies to be determined and accounted for between samples. Our analyses of the effect of Hsp expression on the number of cells with protein inclusions highlight how differences in transfection efficiency between constructs can affect interpretation of the data. Moreover, the Hsp-encoding bicistronic constructs can simultaneously quantify transfection efficiencies and the relative levels of Hsps in live cells by measuring the levels of fluorescence using techniques such as flow cytometry. The fluorescent reporter also provides the option of purifying the transfected cell population by fluorescence-activated cell sorting for subsequent biochemical analyses (i.e. immunoblot, filter-trap, or generation of stable cell lines).

Three different approaches were compared to investigate the effect Hsps have on with the formation of intracellular inclusions, namely analyses that take into account (i) only cells expressing mFluc-EGFP (Fig. 3e); (ii) only co-transfected cells (Fig. 3f); and (iii) only co-transfected cells and the levels of mFluc in those cells (Fig. 3g,h). The latter approach enables assessment of the chaperone efficacy of each Hsp, taking into account the amount of the aggregation-prone protein (in this case mFluc-EGFP) expressed in cells. We believe that this is advantageous as there is a strong correlation between the amount of protein expressed in a cell and its propensity to form inclusions^15, 24–26^. Moreover, in this work, we found that mFluc-EGFP expression levels were influenced by the level of expression from the co-transfected bicistronic construct, whereby reduced expression from one construct resulted in the elevated expression of protein from the co-transfected construct. For example, lower relative levels of expression from the Hsp40- and Hsp90-encoding constructs resulted in significantly higher levels of mFluc in cells (Fig. 3g). This could be reflective of the relative stability of bicistronic mRNA, such that some mRNA are degraded more readily (in this case Hsp40 and Hsp90), which facilitates increased translation of the co-transfected plasmid (in this case mFluc). Thus, we were able to account for differences in the levels of mFLuc-EGFP expression in comparative analyses between samples.

By taking into account the level of mFluc in cells co-transfected to express Hsps, we demonstrate that expression of Hsp70 and/or Hsp40 significantly reduced the proportion of cells with mFluc-EGFP inclusions (Fig. 3h). Whilst the over-expression of Hsps may influence the levels of aggregation-prone proteins in cells (e.g. via promoting their degradation) we did not observe a decrease in mFluc-EGFP levels compared to the EGFP^inv^ control in any of the Hsp-expressing samples (Fig. 3g). Therefore, the reductions observed in the proportion of cells with mFluc-EGFP inclusions can be attributed to Hsps stabilising mFluc to prevent its aggregation, rather than stimulating the degradation of mFluc. Our findings extend on previous studies that have demonstrated the ability of Hsp40 and Hsp70 to refold and resolubilise heat denatured firefly luciferase in simple solution-based assays^27–29^. Furthermore, studies investigating the aggregation of pathogenic proteins such as polyglutamine-expanded huntingtin^15^, human androgen receptor^30^, α-synuclein^31^, and TAR DNA binding protein-43^32^, showed that over-expression of Hsp40 or Hsp70 (and other proteins in these subfamilies) inhibited inclusion formation of each of these client proteins. Hsp40 acts to inhibit protein aggregation by binding misfolded proteins to maintain them in a folding-competent state, delivering the misfolded protein to Hsp70 for active refolding^32^. In addition to actively folding misfolded proteins, Hsp70 can interact with components of the ubiquitin-proteasome system or autophagy to degrade aggregation-prone proteins^33, 34^. Therefore, it would be of great interest to re-evaluate the chaperone activities, particularly the anti-aggregation and cytoprotective roles, of Hsp40 and Hsp70 in the context of disease-associated aggregating proteins using the bicistronic expression constructs developed for this work. Furthermore, we show that increasing levels of Hsp70 or Hsp40 + Hsp70 in cells results in a concentration-dependent decrease in the proportion of cells with mFluc-EGFP inclusions. These findings support the concept of boosting the activity or amount of Hsps in cells as a therapeutic approach to inhibit protein aggregation associated with neurodegenerative diseases^32, 35, 36^.

Interestingly, whilst the sHsps, Hsp27 and αB-c, have been shown to inhibit the aggregation of client proteins and re-fold heat denatured firefly luciferase in solution-based assays, often at sub-stoichiometric levels^37–41^, they did not significantly reduce the proportion of cells with mFluc-based inclusions in this study. A possible reason for this is that these sHsps do not interact with this particular client protein in the context of the cell cytoplasm. It is well-known that the sHsps show some specificity with regards to client proteins with which they can interact. For example, over-expression of Hsp27 no effect on inclusion body formation of the huntingtin exon 1 fragment^42^ but its over-expression significantly reduced the aggregation of α-synuclein^43^. Furthermore, the molecular mechanism by which αB-c inhibits the aggregation of client proteins can vary depending on the stability of the precursor to aggregation^40^. The complexity of sHsp chaperone activity combined with the substantial evidence showing co-localisation between sHsps and protein aggregates in post-mortem brains with neurodegenerative disease^44–48^, indicates that further research is required to establish the roles sHsps play in these diseases with regard to cytoprotection and inhibition of protein aggregation in cells. Bicistronic constructs such as those used here should be useful in such studies.

Studies of Hsp cellular function have typically relied on over-expressing non-labelled Hsps, a strategy generally adopted to minimise any possible adverse effects of tagging a fluorescent protein to these dynamic proteins. In this work we have developed and validated Hsp-encoding bicistronic constructs that provide the correlated expression of a non-labelled Hsp and fluorescent reporter protein. This strategy enables differences in transfection efficiencies and Hsp expression levels (down to the level of individual cells) to be taken into account when performing cell-based assays to investigate and compare the functions of Hsps. This approach can be used in future work to investigate the ability of Hsps to mitigate the underlying molecular mechanisms that characterise a range of neurodegenerative diseases. Moreover, this approach can also be applied to study other proteins whose structure and function are perturbed by fluorescent protein tagging.

## Methods

### Materials

All reagents used for this work were obtained from Sigma-Aldrich (St Louis, MO, USA) or Amresco (Solon, OH, USA) unless otherwise stated. Halt^TM^ Protease and Phosphatase Inhibitor Cocktail (100×) and all restriction enzymes were acquired from Thermo-Fisher Scientific (Scoresby, VIC, Australia). The transfection reagent Lipofectamine® LTX with PLUS^TM^ reagent, 0.025% trypsin-EDTA, Dulbecco’s Modified Eagle Medium/Ham’s F12 media (DMEM/F12), and L-glutamine (100×) were purchased from Invitrogen (Carlsbad, CA, USA). Foetal calf serum (FCS) was obtained from Bovogen Biologicals (Keilor, VIC, Australia).

### Antibodies

Mouse monoclonal anti-Hsp40 (ab78437; 1:5000), anti-Hsp90 (ab13492; 1:5000), anti-Hsp27 (ab2790 1:2500), anti-αB-c (ab13496; 1:5000), anti-mCherry (ab125096; 1:2000) and IgG1-isotype control (ab91353) primary antibodies, and goat anti-mouse IgG DyLight 488 conjugated seconday antibodies (ab96871) were obtained from Abcam (Cambridge, MA, USA). Mouse monoclonal anti-Hsp70 primary antibody (ADI-SPA-810-F; 1:1000) was from Enzo Life Sciences (Farmingdale, NY, USA). Mouse monoclonal anti-α-tubulin primary antibody (T8203; 1:5000) and rabbit polyclonal anti-mouse IgG horse-radish peroxidase conjugated secondary antibody (SAB3701084; 1:5000) were obtained from Sigma Aldrich. Dilutions used for immunoblotting are included in parentheses.

### Plasmids and cloning of Hsp-encoding bicistronic constructs

The pIRES2-EGFP plasmid was obtained from Clontech (Palo Alto, CA, USA). A series of constructs were generated from the pIRES2-EGFP plasmid that transcribes bicistronic mRNA consisting of an IRES flanked by an upstream Hsp and downstream fluorescent reporter. The mCherry gene (GenBank AY678264) was synthesised by GenScript with flanking 5′ *Bst*XI and 3′*Not*I restriction sites to allow replacement of the EGFP in pIRES2-EGFP with mCherry to generate pIRES2-mCherry. Primers were designed to amplify genes encoding Hsps (with flanking restriction sites) from existing plasmid constructs for sub-cloning upstream of the IRES site of the pIRES2 plasmids; αB-crystallin (*CRYAB*; GenBank NM_001885) with *Nhe*I/*Sal*I, HSP27 (*HSPB1*; GenBank BT019888.1) with *Bgl*II/*Sal*I, HSP70 (*HSPA1A*; GenBankAK291295.1; gifted by Prof Sophie Jackson, Cambridge University, UK) with *Nhe*I/*Bam*HI. Genes encoding HSP40 (*DNAJ1*; GenBank NM_001539.2) with *Nhe*I/*Bam*HI sites, and HSP90 (HSP90AA1; GenBank NM_001017963.2) with *Sal*I/*Not*I sites, were synthesised by GenScript, prior to their digestion from the supplied pUC57 constructs and sub-cloning into pIRES2-mCherry. In addition to the Hsp-encoding bicistronic constructs, a plasmid was constructed with flanking *Bgl*II/*Eco*RI sites to encode for EGFP^inv^ ^49^, a non-fluorescent derivative of GFP, which is in place of a HSP and acted as a chaperone-negative control. All of the constructs synthesised in this work were verified by sequencing using a Hitachi 3130*xl* Genetic Analyser (Applied Biosystems, Mulgrave, Australia).

Mammalian expression constructs containing sequences encoding wild-type and the conformationally destabilised double mutant of firefly luciferase-EGFP (WT Fluc-EGFP,pcDNA4-TO-myc-hisA-Fluc WT and mFluc-EGFP, pcDNA4-TO-myc-hisA-Fluc R188Q/R261Q respectively) were a kind gift from Prof Mark Wilson (University of Wollongong).

### Neuro-2a cell culture and transfection

Neuro-2a cells were obtained from the American Type Culture Collection (Manassas, VA, USA). Cells were cultured in Dulbecco’s Modified Eagle Medium and Ham’s nutrient mixture F-12 supplemented with 2.5 mM L-glutamine and 10% (v/v) FCS (10% FCS-DMEM/F-12) at 37 °C under 5% CO_2_/95% air in a Heracell 150i CO_2_ incubator (Thermo Fisher Scientific). Cells were passaged every 2 days or once they had reached 80% confluency and routinely tested for mycoplasma contamination.

For transfections, 7.5 × 10^4^ cells/mL were seeded (unless otherwise stated) into a 6-well plate and cultured in 2 mL of 10% FCS DMEM/F-12 overnight. These cells were transiently transfected with the bicistronic vectors using Lipofectamine LTX/PLUS reagent. Cells were transfected with DNA:lipid complexes (2 μg/well of DNA, 6 μL/well of Lipofectamine LTX and 2 μL/well PLUS™ reagent) and incubated for 48 h at 37 °C under 5% CO_2_/95% air. The cells were harvested with trypsin 48 h post-transfection, washed twice with PBS (pH 7.4) and either fixed in 4% (w/v) paraformaldehyde in PBS at room temperature (RT) for 30 min, or live transfected cells were purified by fluorescence-activated cell-sorting for subsequent analyses.

### Immunocytochemistry and confocal microscopy

A 12-well plate containing sterile 19 mm coverslips (ProSciTech, Thuringowa, Australia) was seeded with 4.0 × 10^4^ cells/well and cultured in 10% FCS-DMEM/F-12 overnight at 37 °C under 5% CO_2_/95% air. Cells were transfected with 1 µg/well of pIRES2-EGFP-αB-c DNA and 1.5 μLLipofectamine® LTX with 0.5 μL PLUS™ reagent, and incubated for 48 h at 37 °C under 5% CO_2_/95% air. Cell culture media was removed 48 h post-transfection and coverslips were washed twice with PBS (pH 7.4). Cells were fixed by incubation with 4% (w/v) paraformaldehyde (PFA) for 20 min at RT, washed twice with PBS, and permeabilised by incubation with 0.5% (v/v) Triton X-100 in PBS for 20 min at room temperature. Coverslips were washed twice with 1% (w/v) BSA in PBS, blocked for 30 min at room temperature using 5% (w/v) BSA in PBS, and washed twice in antibody incubation buffer (1% (w/v) BSA in PBS-Tween 20; 1 × PBS containing 0.05% (v/v) Tween-20). Intracellular Hsps were immunolabelled by overlaying coverslips with anti-αB-c primary antibody (1:500) and incubating for 1 h at 37 °C in a humidity chamber. Cells were also incubated with a species-matched IgG isotype control primary antibody (1:500) to account for background staining in immunolabelled cells. After three washes in antibody incubation buffer, coverslips were incubated with DyLight 488-conjugated anti-mouse IgG secondary antibody (1:500), for 30 min at 37 °C in a humidity chamber. Coverslips were washed three times with in antibody incubation buffer before mounting on microscope slides for confocal microscopy.

Immunolabelled coverslips were mounted onto 26 × 76 mm glass slides (Thermo Fisher Scientific) using Citifluor™ Anti-Fadent Mounting Solutions (ProSciTech). The slides were analysed using a Leica TCS SP5 confocal microscope using the ×60 oil-immersion objective lens (Leica Microsystems, Wetzlar, Germany). Fluorescence was excited at 488 nm and 561 nm by argon and DPS 561 lasers, respectively. Fluorescent emissions from EGFP and DyLight 650 were acquired by sequential scanning using the Leica Application Suite – Advanced Fluorescence (LAS-AF) software (version 3, Leica Microsystems, Wetzlar, Germany).

### Cell preparation for sorting

To purify mCherry^+ve^ cells for subsequent immunoblotting, cells transfected with the Hsp-encoding bicistronic constructs were harvested with trypsin 48 h post-transfection. Samples were washed twice in PBS (pH 7.4; 5 min, 300 × *g*) and resuspended in fluorescence-activated cell sorting (FACS) buffer (25 mM HEPES, 1 mM EDTA, 0.5% w/v bovine serum albumin in PBS, pH 7.0). Cell clumps were removed by straining through a 40 µm nylon mesh before analysis on an S3e Cell Sorter equipped with a 561-nm laser (Bio-Rad Laboratories, Hercules, CA, USA). mCherry^+ve^ cells were sorted such that 300,000 cells were recovered.

To confirm that cells resolved in the iPop did indeed have inclusions, cells were transfected to express mFluc-EGFP and fixed in 1% (w/v) PFA in PBS (pH 7.4) for 30 min on ice. Samples were washed twice in PBS (5 min, 300 × *g*) and resuspended in FACS buffer. Cells were sorted on a FACSAriaII equipped with a 488-nm laser (BD Biosciences, San Jose, CA, USA) at the MWAC BRIL Flow Cytometry Facility, University of New South Wales.

### Immunoblotting

FACS-purified populations of mCherry^+ve^ cells were collected (10 min, 1000 × *g*) and subsequently immunoblotted for Hsp and mCherry expression in samples transfected with each of the Hsp-encoding bicistronic constructs. Whole cell lysates were prepared in SDS extraction buffer (2% w/v SDS in 0.5 M Tris-HCl, 1 × protease/phosphatase inhbitors) and heated (5 min, 95 °C). Prior to loading onto the gel, samples were mixed with SDS loading buffer (2% w/v SDS, 0.5 M Tris-HCl, 5% v/v glycerol, 0.01% w/v bromophenol blue containing 5% v/v β-mercaptoethanol) and heated (95 °C/5 min). Whole cell lysates corresponding to 100,000 cells per well were loaded on 12% (v/v) polyacrylamide gels and resolved by SDS-PAGE following standard procedures^50^. Resolved proteins were electroblotted onto polyvinylidene difluoride membranes (Bio-Rad Laboratories) using standard techniques^51^. Membranes were blocked with 5% (w/v) non-fat milk in TBS-T (Tris buffered saline; 50 mM Tris and 150 mM NaCl, 0.05% v/v Tween-20, pH 7.4) for 1 h at RT and incubated at 4 °C for 16 h with 5% (w/v) non-fat dry milk in TBS-T and primary antibodies. The blots were washed four times (10 min) with TBS-T and incubated for 1 h in 5% (w/v) non-fat dry milk in TBS-T with horseradish peroxidase-conjugated rabbit anti-mouse secondary antibody. Detection of binding was determined using Super Signal West Pico Chemiluminescent Substrate (Thermo Fisher Scientific) and exposure to Amersham Hyperfilm ECL (GE Healthcare Life Sciences, Uppsala, Sweden). Images presented in Fig. 1 have been cropped and full length blots are presented in Fig. S2.

### Immunolabelling of Hsps in transfected Neuro-2a cells for flow cytometry

Fixed cells were permeabilised by incubation with 0.5% (v/v) Triton X-100 in PBS at 4 °C for 30 min. Cells were washed twice (400 × *g*/15 min) with 0.1% (w/v) BSA in PBS, then blocked with 3% (w/v) BSA in PBS for 30 min, before incubating with primary antibodies (anti- Hsp40/Hsp70/Hsp90/Hsp27/αB-c or IgG1-isotype control; 1 μg/10^6^ cells) for 30 min at RT with gentle rocking. The cells were washed twice with 0.1% (w/v) BSA in PBS and the cell pellets were resuspended in 3% (w/v) BSA in PBS. The cells were incubated with secondary antibody (goat anti-mouse IgG conjugated to DyLight 488; 1:200 dilution) for 30 min at RT with rocking, washed twice with PBS and then analysed using an LSRFortessaX-20 cell analyser equipped with 488- and 561-nm lasers (BD Biosciences, San Jose, CA, USA).

### Cell-based model of protein aggregation: Mutant firefly luciferase (mFluc-EGFP)

A 6-well plate was seeded with 2.0 × 10^6^ Neuro-2a cells/well and maintained in 10% FCS-DMEM/F-12 overnight at 37 °C under 5% CO_2_/95% air. Cells were co-transfected with the mFluc-EGFP encoding constructs (1.25 µg) and one of the Hsp-encoding (or EGFP^inv^) pIRES2-mCherry constructs (0.25 µg), such that cells were transfected with a 5:1 (mFluc-EGFP:Hsp) ratio of each construct. Each DNA construct was incubated in separate tubes with Lipofectamine LTX and PLUS reagent according to the manufacturer’s instructions. The DNA:lipid complexes were sequentially applied to the cells. Cells were harvested with trypsin 48 h post-transfection, washed twice in ice-cold PBS (300 × *g*, 5 min, 4 °C) and resuspended in 500 μL ice-cold PBS for analysis by flow cytometry.

Some cells were left untransfected or only transfected with mFluc-EGFP- or EGFP^inv^ encoding constructs. These samples were used to set gates and to determine the spectral overlap that occurs between mCherry and EGFP fluorescence emissions in this experiment using the compensation matrix in Flow Jo (version 10.0.8, Tree Star, Ashland, OR, USA). The spectral overlap was negligible (0.0028% spectral overlap) in these experiments.

The relative EGFP fluorescence was used to represent mFluc levels in the cell (Fig. 3h). The data presented was analysed using equation 1:

1 $$Relative\,EGFP\,fluorescence=\frac{EGFP\,fluorescent\,median\,of\,sample\,x}{EGFP\,fluorescent\,median\,of\,sample\,EGFPinv}$$

The percent of cells in the iPop gate in each sample was normalised to the relative EGFP fluorescence in that sample. In this way, differences in the relative levels of mFluc-EGFP expression were taken into account and data was analysed using equation 2:

2 $$ \% \,cells\,in\,iPop\,normalised\,to\,mFluc\,levels=\frac{ \% \,cells\,in\,iPop\,of\,sample\,x}{Relative\,EGFP\,fluorescence\,of\,sample\,x}$$

### Flow cytometry

Flow cytometry was performed using an LSRFortessaX-20 cell analyser equipped with 488-nm and 561-nm lasers (BD Biosciences). A minimum of 20,000 events per sample were collected at a high flow rate. Forward scatter was collected using a linear scale and side scatter in a log scale. Fluorescent emissions were collected as area (log scale), pulse height (log scale), and pulse width (linear scale) for each channel. For mCherry fluorescence, data were collected with the 561-nm laser and 586/15 bandpass filter. For EGFP and DyLight 488, data were collected with the 488-nm laser and 525/50 bandpass filter. Flow cytometric gating and data analysis was performed using Flow Jo software (Tree Star).

### Statistics

Results shown are the mean ± S.E.M. of three independent experiments. Statistical analyses were performed using GraphPad Prism 5 (GraphPad Software, Inc., La Jolla, CA, USA). With respect to binning of mCherry fluorescence into 16 bins of equal RFU, bins containing less than 100 cells were excluded from subsequent analysis. Evaluation of differences in means was determined by a student’s t-test or a one-way analysis of variance (ANOVA) for multiple comparisons. The F-statistic from the one-way ANOVA test and its associated degrees of freedom (between groups and within groups, respectively) are reported in parentheses. The *p*-value from the one-way ANOVA test is also stated. Post hoc testing of differences between means was done using Dunnett’s test, where an alpha level of 0.05 was considered significant.

## Electronic supplementary material

Supplementary info
