# Supplementary material for: Using bicistronic constructs to evaluate the chaperone activities of heat shock proteins in cells
Source: Sci Rep. 2017 May 24;7:2387. doi: 10.1038/s41598-017-02459-9 (PMC5443837; doi:10.1038/s41598-017-02459-9)
Supplement: Supplementary file 1 — Supplementary info [file 41598_2017_2459_MOESM1_ESM.pdf]

**Using bicistronic constructs to evaluate the chaperone activities of heat shock proteins in cells**

Rebecca San Gil[1,2], Tracey Berg[1], Heath Ecroyd[1,2]

[1]Illawarra Health and Medical Research Institute, University of Wollongong, Northfields Ave, Wollongong, 2522, Australia

[2]School of Biological Sciences, University of Wollongong, Northfields Ave, Wollongong, 2522, Australia

heathe@uow.edu.au; Ph: +61 242213443

**Supplementary material**

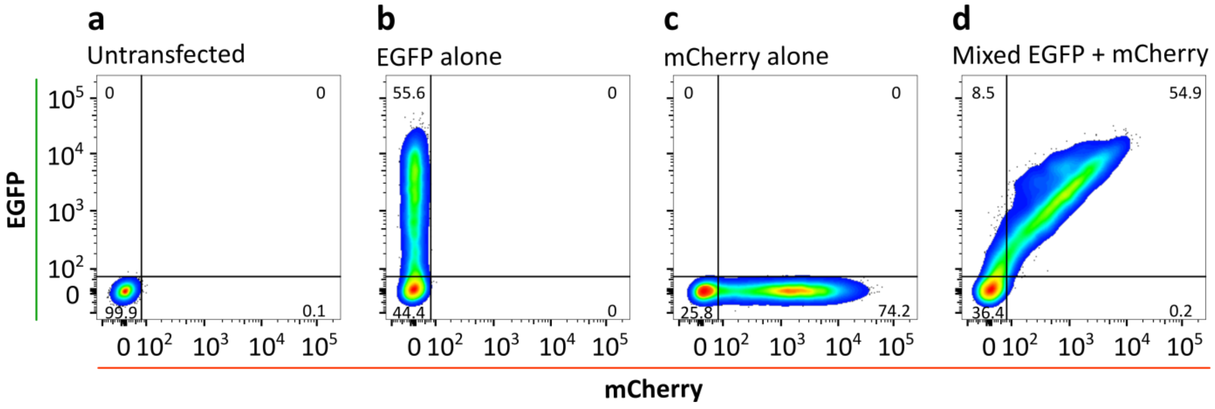


**Supplementary Figure S1. Co-transfection of cells with two constructs as an alternative approach to using bicistronic constructs.** Neuro-2a cells were either (**a**) untransfected or (**b**) co-transfected with pIRES2-EGFP$^{inv}$-EGFP and pIRES2-EGFP$^{inv}$-mCherry by mixing the two constructs together with transfection reagent. The co-transfected cells (55.3%) showed a high correlation between EGFP and mCherry expression (e.g. cells with high expression levels of EGFP also show high mCherry fluorescence).

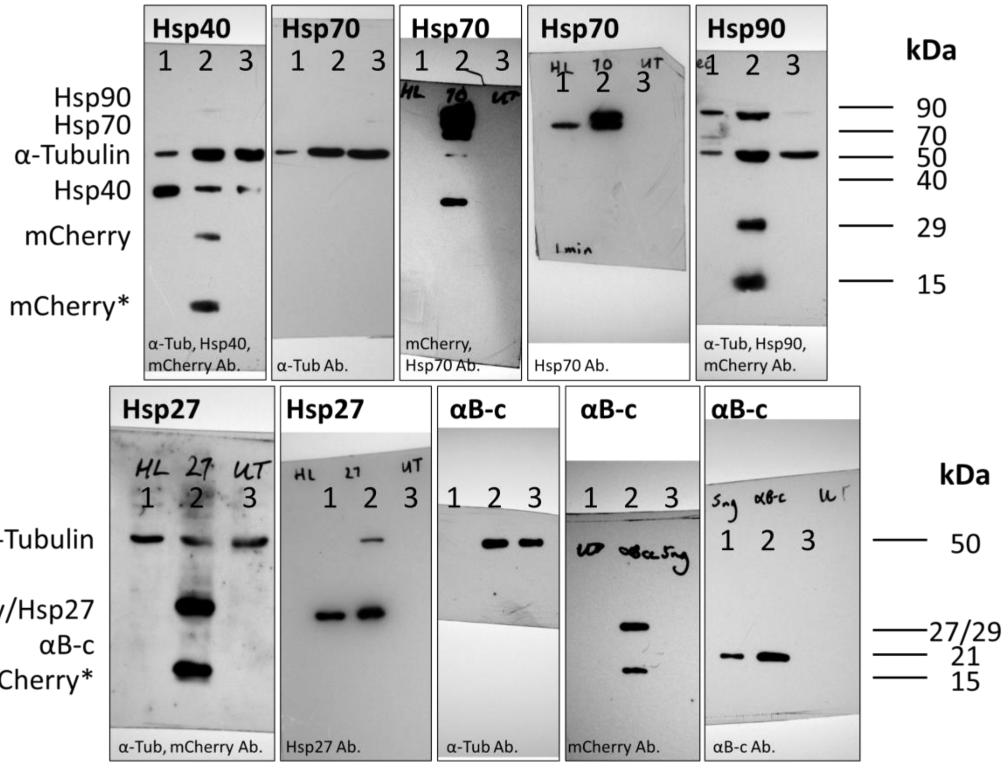


**Supplementary Figure S2. Whole blots from immunoblot analysis of Hsp and mCherry in Neuro-2a cells transfected with each of the Hsp-encoding pIRES2 bicistronic constructs (Figure 1).** Neuro-2a cells transfected with each of the Hsp-encoding bicistronic constructs were sorted by FACS such that a population of mCherry$^{+ve}$ cells were purified and whole cell lysates equivalent to 100,000 cells were loaded into each well. The membranes were probed for α-tubulin (50 kDa), mCherry (29 kDa) (mCherry* = degraded fragment) and Hsp40, Hsp70, Hsp90, Hsp27, and αB-c. The specific combination of antibodies (Ab.) used on each blot is listed below each blot. Protein samples analysed were (1) 10 µg of heat-shocked HeLa (HL) cell lysate (42°C/2 h with a 37°C/3 h recovery period) or 5 ng purified recombinant αB-c for blots probing for αB-c, (2) whole cell lysates from Neuro-2a transfected with each of the Hsp-encoding bicistronic constructs, and (3) untransfected (UT) cells. Labels within the blots denotes which of the Hsp-encoding bicistronic constructs Neuro-2a cells in lane 2 have been transfected with.

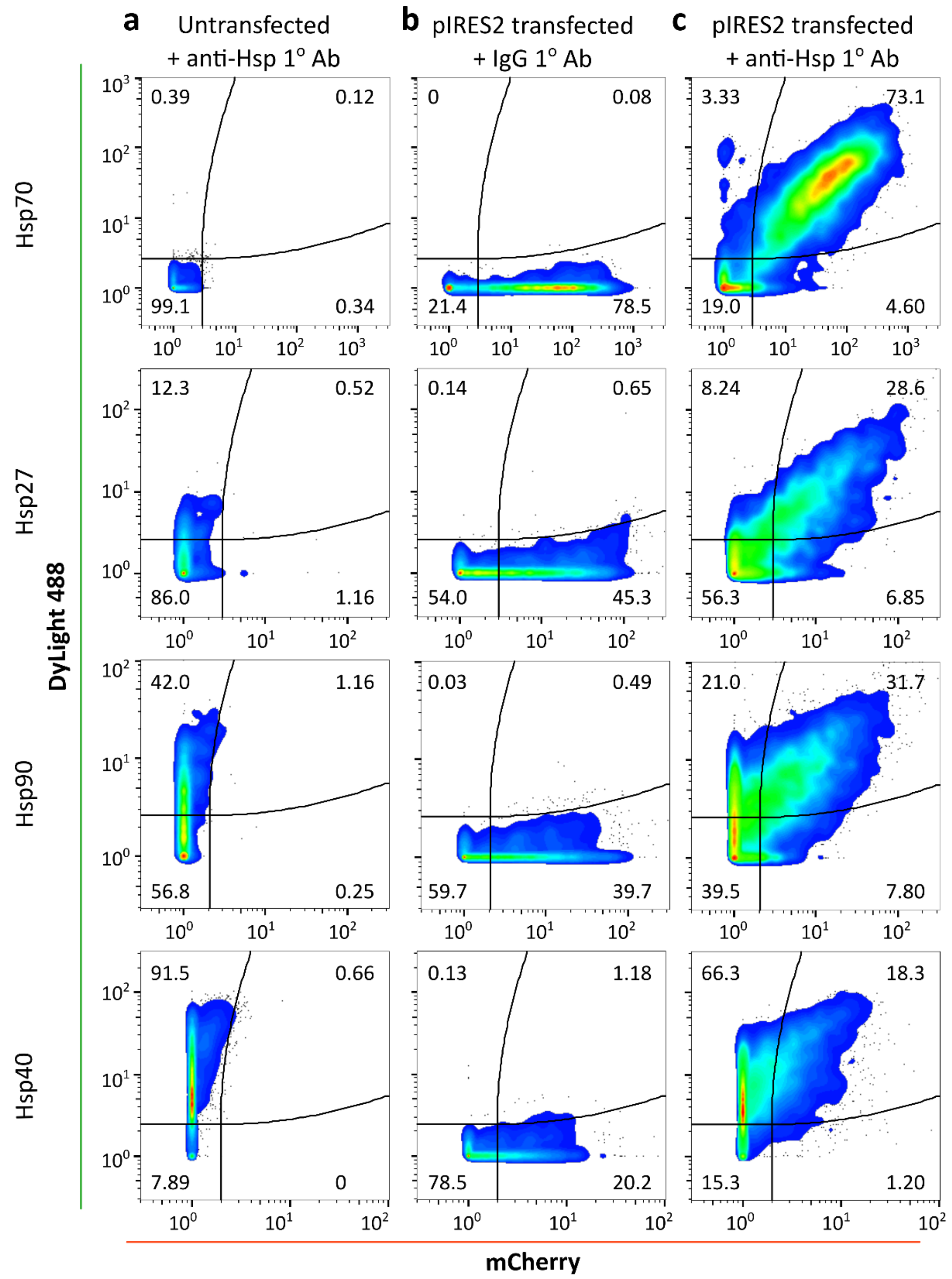


**Supplementary Figure S3. Flow cytometric verification of the correlated expression of mCherry and each of the Hsps in individual cells following transfection of cells with each of the Hsp-encoding bicistronic constructs.** Flow cytometric data are presented as pseudo-colour plots where blue depicts – low, green – medium and red – high frequency of cells. Outliers are shown as black dots. Cellular debris and cell clumps were excluded based on forward and side scatter (see Figure 2a) such that subsequent analysis was only performed on viable cells. Quadrant gating of DyLight 488 and mCherry fluorescence was based on untransfected and unlabelled cells (see Figure 2b). (**a**) Untransfected cells were immunolabelled with anti-Hsp40/Hsp70/Hsp90/Hsp27/αB-c primary (1°) and DyLight 488 conjugated secondary antibodies. (**b**) Cells were transfected with each of the Hsp-encoding bicistronic constructs and immunolabelled with species-matched IgG control to account for background primary antibody staining. (**c**) Cells were transfected with each of the Hsp-encoding bicistronic constructs and subsequently immunolabelled with the respective anti-Hsp40/Hsp70/Hsp90/Hsp27/αB-c primary and DyLight 488 conjugated secondary antibodies. Note the scale across the cytograms in this figure varies between panels.

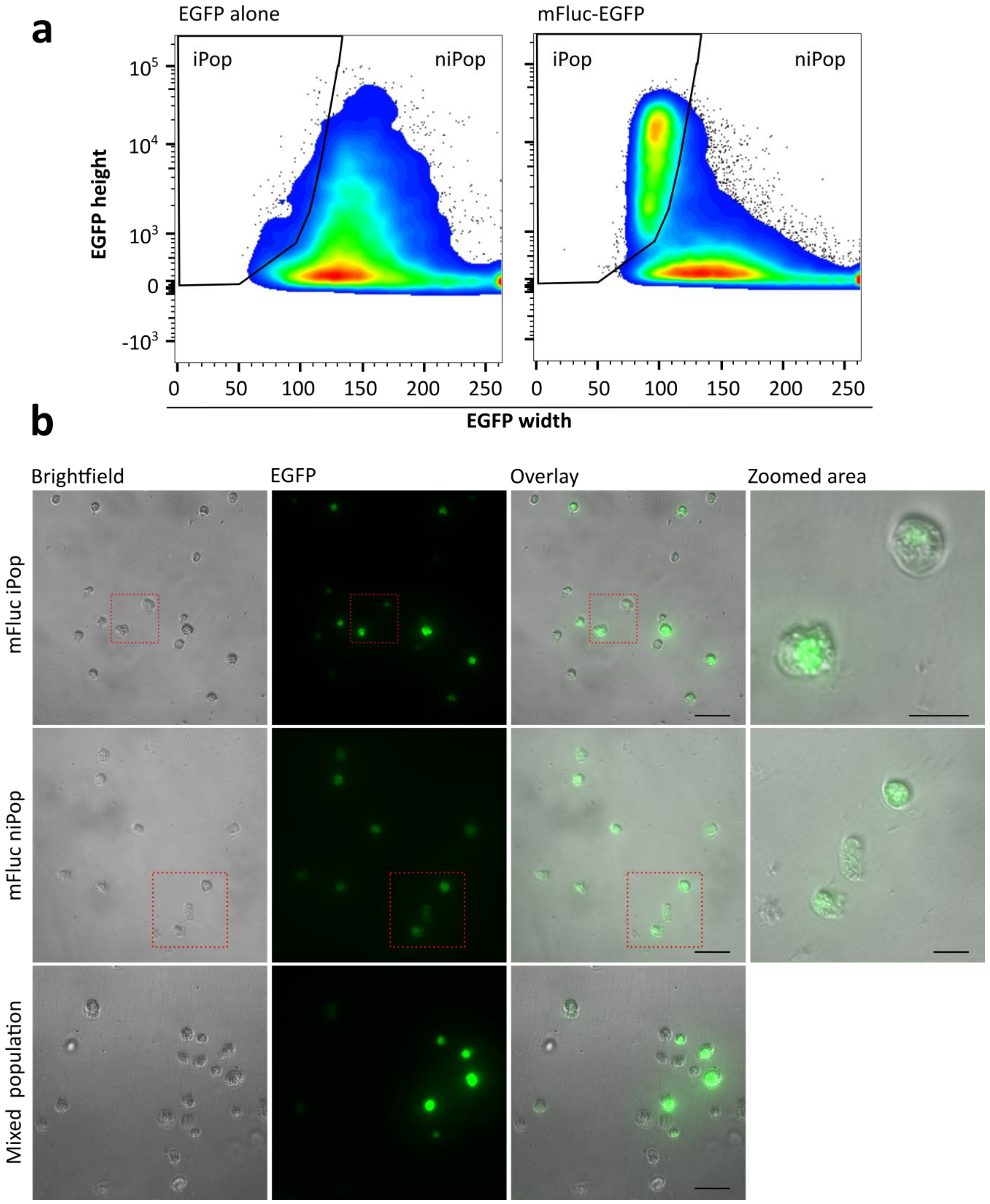


**Supplementary Figure S4. Fluorescence activated cell sorted iPop and niPop populations.** Neuro-2a cells were transfected to express either EGFP alone or mFluc-EGFP, fixed 48-hours post-transfection and sorted for fluorescence microscopy. (**a**) PulSA of cells expressing EGFP alone (*left*) and mFluc-EGFP (*right*). Cells expressing EGFP alone were used to set the iPop polygonal gate. (**b**) Representative images are shown of the sorted iPop, niPop and the mixed cell population prior to FACS. *Left – right:* brightfield images, EGFP fluorescence, the overlay and a zoomed region of interest denoted by the red dashed square. Scale bar = 50 µm and 20 µm in the zoomed images. The cell sorting experiment was designed to confirm that cells resolved by PulSA in the iPop did indeed have mFluc-EGFP inclusions. We show here that cells with mFluc-EGFP inclusions were exclusively found in the iPop sorted cells and cells with soluble mFluc-EGFP in the niPop, indicating that PulSA is an appropriate technique to resolve cells with mFluc-EGFP inclusions.

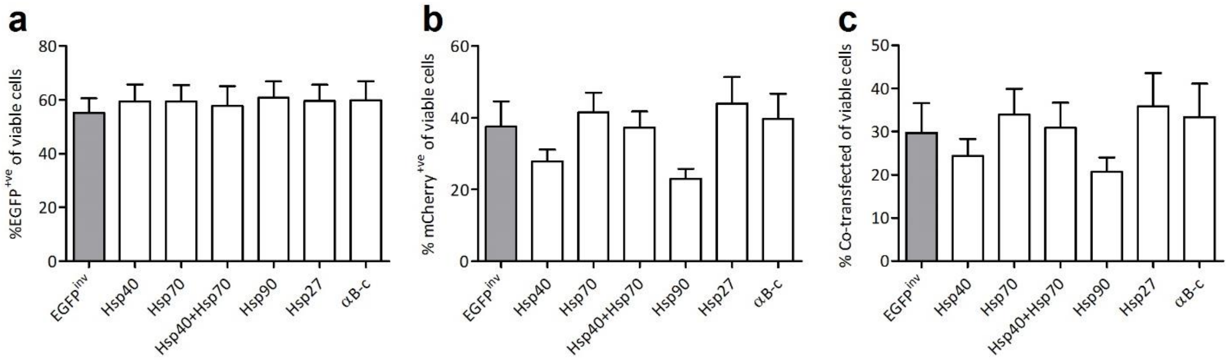


**Supplementary Figure S5. Bicistronic constructs enable easy determination of transfection and co-transfection efficiencies by flow cytometry.** (**a**) The percent of $EGFP^{+ve}$ cells of the viable cell population. (**b**) The percent of $mCherry^{+ve}$ cells of the viable cell population. (**c**) The percent of co-transfected cells (ie. $EGFP^{+ve}$ and $mCherry^{+ve}$) of the viable cell population. Data presented are the means ± SEM of three biological replicates.

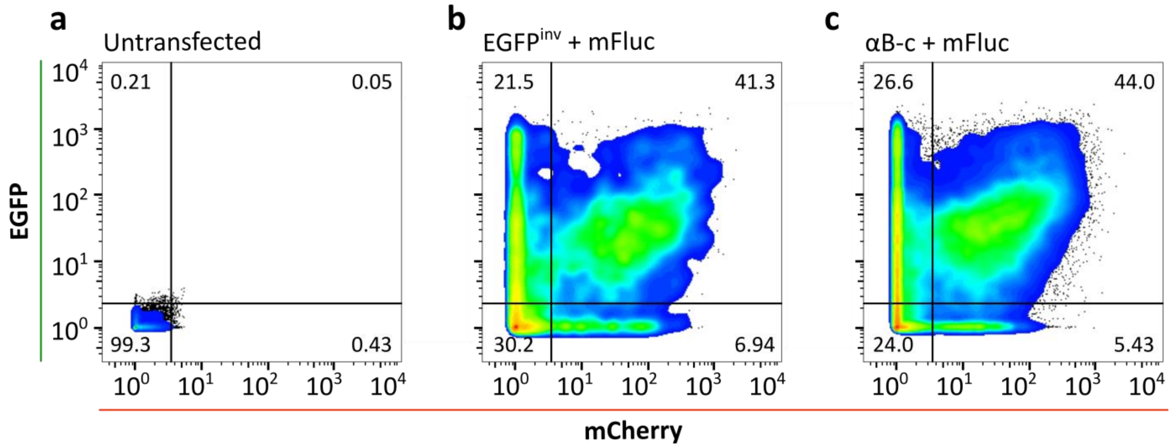


**Supplementary Figure S6. Co-transfections in Neuro-2a cells for the mFluc-EGFP cell-based model of protein aggregation.** With regards to analyses involving mCherry binning, we attempted to overcome the likelihood that cells expressing high levels of Hsp also express high levels of mFLuc by preparing the constructs separately for transfection and applying them to the cells sequentially. In this way, cells were randomly either, singly transfected, co-transfected, or untransfected. Whilst this methodology for the co-transfections still resulted in a weak correlation between Hsp and mFluc expression, it resulted in a greater range in the expression of both plasmids compared to the method involving mixing the plasmids prior to making the DNA:lipid complexes (see Fig. S1). We also took the relative levels of mFLuc expression into account when investigating the effect of increasing Hsp levels on the proportion of cells with inclusions by dividing the proportion of cells with inclusions by the median mFluc-EGFP fluorescence intensity (Fig. 4). Representative cytograms are shown of Neuro-2a that were either (**a**) untransfected, or co-transfected to express (**b**) $EGFP^{inv}$ with mCherry and mFluc-EGFP and (**c**) αB-c and mFluc-EGFP. Quadrant gating was based on the untransfected sample and the proportion of cells in each quadrant is shown.

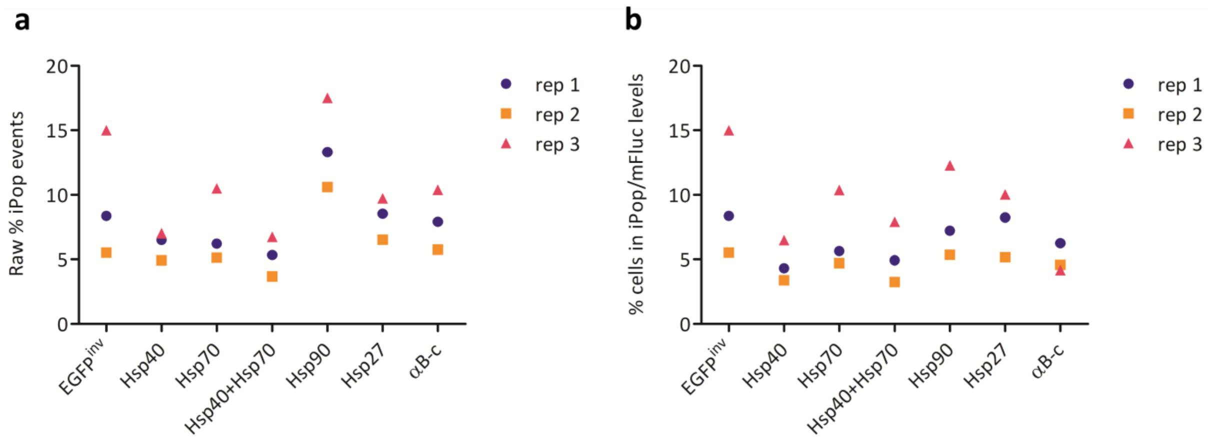


**Supplementary Figure S7. Graphs of raw percent of cells in the iPop gate.** Cells were co-transfected with mFluc-EGFP and each of the Hsp-encoding bicistronic constructs and 48 h post-transfection the effect of each expressing each Hsp on mFluc inclusion formation was analysed by flow cytometry. (**a**) Raw percent of cells in the iPop gate within each sample across each of the three biological replicates, ● rep 1, ■ rep 2 and ▲ rep 3. (**b**) The percent of cells in the iPop gate normalised to relative mFluc expression levels in each respective sample across the three biological replicates. We found that the raw percent of cells with inclusions varied between biological replicates, for example, the third biological replicate demonstrated a consistently higher proportion of cells with inclusions across all samples. However, despite the differences in the raw percent of cells in the iPop gate, the trends across the Hsp expressing samples remained constant between experiments. Therefore, the data shown in Fig. 3 is expressed as a fold change relative to the EGFP$^{inv}$ sample rather than a raw percent to better reflect the chaperone activities of each Hsp in the mFluc protein aggregation assay.
